# Sodium intake and the risk of various types of cardiovascular diseases: a Mendelian randomization study

**DOI:** 10.3389/fnut.2023.1250509

**Published:** 2023-12-22

**Authors:** Qingming Fu, Rumeng Chen, Yining Ding, Shuling Xu, Chunxia Huang, Binsheng He, Ting Jiang, Bin Zeng, Meihua Bao, Sen Li

**Affiliations:** ^1^School of Stomatology, Changsha Medical University, Changsha, China; ^2^Hospital of Chengdu University of Traditional Chinese Medicine, Chengdu, China; ^3^School of Life Sciences, Beijing University of Chinese Medicine, Beijing, China; ^4^The Second Affiliated Hospital of Anhui Medical University, Heifei, China; ^5^The Hunan Provincial Key Laboratory of the TCM Agricultural Biogenomics, Changsha Medical University, Changsha, China; ^6^Hunan Key Laboratory of the Research and Development of Novel Pharmaceutical Preparations, School of Pharmaceutical Science, Changsha Medical University, Changsha, China

**Keywords:** sodium intake, cardiovascular disease, Mendelian randomization, UK Biobank, FinnGen

## Abstract

**Background:**

The existing literature on the link between sodium intake and cardiovascular disease (CVD) largely consists of observational studies that have yielded inconsistent conclusions. In this study, our objective is to assess the causal relationship between sodium intake and 50 CVDs using two-sample Mendelian randomization (MR) analysis.

**Methods:**

MR analyses were performed to investigate the associations between urinary sodium/creatinine ratio (U_Na_/U_Cr_), an indicator of sodium intake, and 50 CVDs. The genome-wide association study (GWAS) for U_Na_/U_Cr_ was from the UK Biobank (UKBB), and the GWASs for CVDs were from FinnGen. A false discovery rate (FDR) threshold of 5% was applied for multiple comparison correction.

**Results:**

The inverse-variance weighted method indicated that the genetically predicted U_Na_/U_Cr_ was significantly associated with 7 of 50 CVDs, including “Coronary atherosclerosis” (OR = 2.01; 95% CI: 1.37, 2.95), “Diseases of arteries, arterioles and capillaries” (OR = 1.88; 95% CI: 1.20, 2.94), “Hard cardiovascular diseases” (OR = 1.71; 95% CI: 1.24, 2.35), “Ischemic heart diseases” (OR = 2.06; 95% CI: 1.46, 2.93), “Major coronary heart disease event” (OR = 1.99; 95% CI: 1.36, 2.91), “Myocardial infarction” (OR = 2.03; 95% CI: 1.29, 3.19), and “Peripheral artery disease” (OR = 2.50; 95% CI: 1.35, 4.63). Similar results were obtained with the MR-Egger and weighted median methods. No significant heterogeneity or horizontal pleiotropy was found in this analysis.

**Conclusion:**

Our study has uncovered a significant positive causal relationship between U_Na_/U_Cr_ and various CVDs. These results offer a new theoretical foundation for advocating the restriction of sodium intake as a preventive measure against CVD.

## Introduction

Cardiovascular disease (CVD) ranks as the primary global cause of mortality (1–3), accounting for approximately 32% of all mortality cases (4). Since 1990, the number of individuals affected by CVD has doubled, and as of 2019, it is estimated that approximately 523 million individuals have suffered from CVD worldwide (5). Furthermore, CVD is associated with a high global disability rate, placing a significant burden on both patients and their families (6, 7).

The observational studies have indeed shown that both excessive and insufficient salt intake can elevate the risk of cardiovascular disease and mortality (8–10). Accurate assessment of sodium intake is crucial, but direct measurement of sodium intake levels in clinical settings is challenging. Several studies have shown that the urinary sodium/creatinine ratio (U_Na_/U_Cr_) is a reliable indicator and is a simpler, more accessible approach for sodium intake assessment (11–13). However, previous observational research on the association between U_Na_/U_Cr_ and the risk of CVD has yielded inconsistent results. A cross-sectional study carried out in South Korea found a correlation between U_Na_/U_Cr_ and hypertension (14), whereas two other studies in Chinese populations did not find any association between U_Na_/U_Cr_ and hypertension (15, 16). Observational studies are limited in their ability to establish causal relationships, and causal studies are needed to investigate the relationship between U_Na_/U_Cr_ and CVDs.

Mendelian randomization (MR) analysis is an epidemiological approach that employs genetic variation closely linked to the exposure of interest as an instrumental variable (IV). The methodologies used in MR analysis are based on Mendel's second law, which states that alleles follow a principle of random allocation. This property assists in mitigating biases arising from confounding factors and reverses causation. In order to investigate if high levels of U_Na_/U_Cr_ can result in numerous cardiovascular diseases (including 50 types of CVDs), this study will apply MR analyses.

## Methods

### Study design

Single-nucleotide polymorphisms (SNPs) are the most common genetic variations used as IVs in MR, which are employed to calculate the causal associations of traits with diseases. In this study, we performed MR analyses employing data from GWAS datasets for both exposure (U_Na_/U_Cr_) and outcome (50 CVDs).

### Data sources

In the MR analysis, we used a variety of publicly available GWAS summary data (Supplementary Table 1). To fulfill the requirements of the two-sample MR design, the exposure and outcome were obtained from two different European populations, as described previously (17).

Previous studies have shown that estimates obtained from spotted urine samples are effective in assessing 24-h urinary sodium excretion. This effectiveness can be achieved by establishing equations that take into account the U_Na_/U_Cr_. Because sodium intake and excretion are correlated, U_Na_/U_Cr_ was used as a measure of sodium intake. This approach corrected for variations in urinary concentration due to differences in fluid intake levels, making it a more precise indicator of sodium intake. The GWAS for U_Na_/U_Cr_ (sample size = 327,616) was obtained from the UK Biobank (UKBB) (18). The UKBB enlisted half a million individuals, aged 40–69, from various regions of the country to participate in this initiative between 2006 and 2010. These participants have undergone assessments, submitted samples of blood, urine, and saliva for future analysis, provided comprehensive personal information, and consented to continuous health monitoring.

We selected the GWASs for CVDs from the FinnGen datasets, a large-scale biomedical research project based in Finland (19). The FinnGen dataset consisted of genomic data collected from 500,000 individuals of Finnish ancestry. This dataset was then merged with data obtained from the National Healthcare Register of Finland. During the selection process, we excluded a series of phenotypes, such as (1) phenotypes that are not related to the circulation system; (2) similar phenotypes but with a smaller sample size or particular population; (3) broad-defined phenotypes that cannot be specifically categorized as a certain disease; and (4) phenotypes that involved interventions such as operations and medications. As a result, we retained 50 CVDs as outcomes for this study. The corresponding sample sizes for each CVD are provided in Supplementary Table 1.

### Statistical method

In the MR analysis, IVs were selected according to a set of criteria, including: (1) IVs and exposure were significantly associated at the genomic level (*P* < 5.00E-08); (2) independent IVs identified by clumping within a 10 Mb window and linkage disequilibrium (LD) of R^2^ < 0.001; and (3) the minor allele frequency (MAF) > 0.01. Additionally, we excluded palindromic SNPs with an intermediate allele frequency, as previously reported (20). The F-statistics were calculated for the strength of IVs, and a value over 10 indicates a lower risk of weak IV bias (Supplementary Table 2) (21).

The primary strategy utilized in the MR analysis was the inverse-variance weighted (IVW) method. Additionally, we used the weighted median (WM) and MR-Egger methods in sensitivity analyses. The WM method remains unbiased under the condition that no more than 50% of the weight comes from invalid instruments. The pleiotropy-corrected data from MR-PRESSO were used to eliminate probable outliers. The Cochrane Q-value was employed to assess heterogeneity. With the leave-one-out strategy, which examined how each included IV affected the causal associations, the robustness of the results was assessed. Causal estimations were presented as odds ratios (ORs) and 95% confidence intervals (CIs). To address multiple comparisons, a false discovery rate (FDR) threshold of 5% was applied for correction. The two-sample MR package was used to perform all the MR analyses in R.

## Results

**Figure 1:**
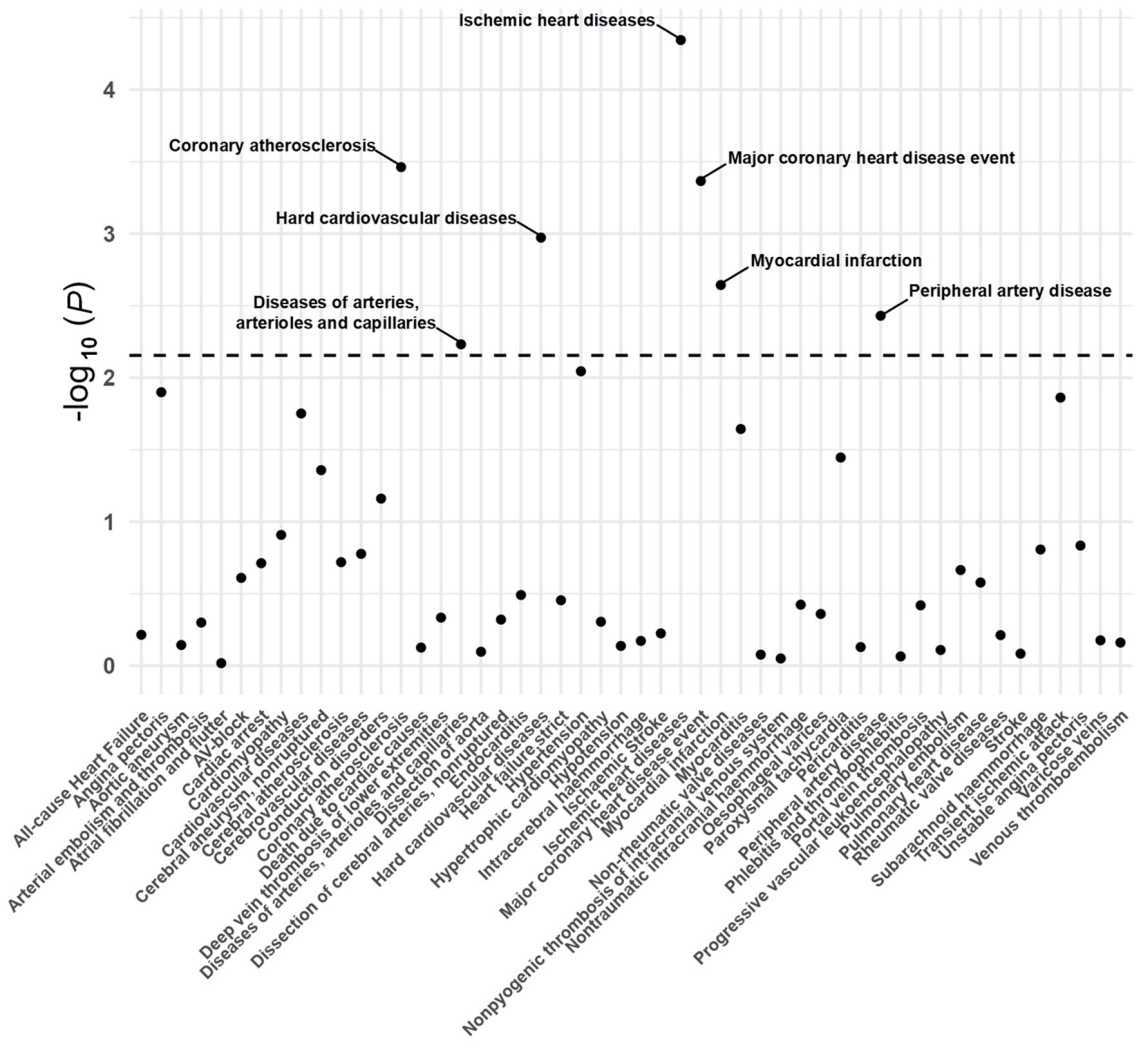
*P*-value distribution of associations between urinary sodium/creatinine ratio (U_Na_/U_Cr_) and 50 CVDs in the Mendelian randomization analysis. The dashed line represents the significance threshold adjusted by the false discovery rate.

**Figure 2:**
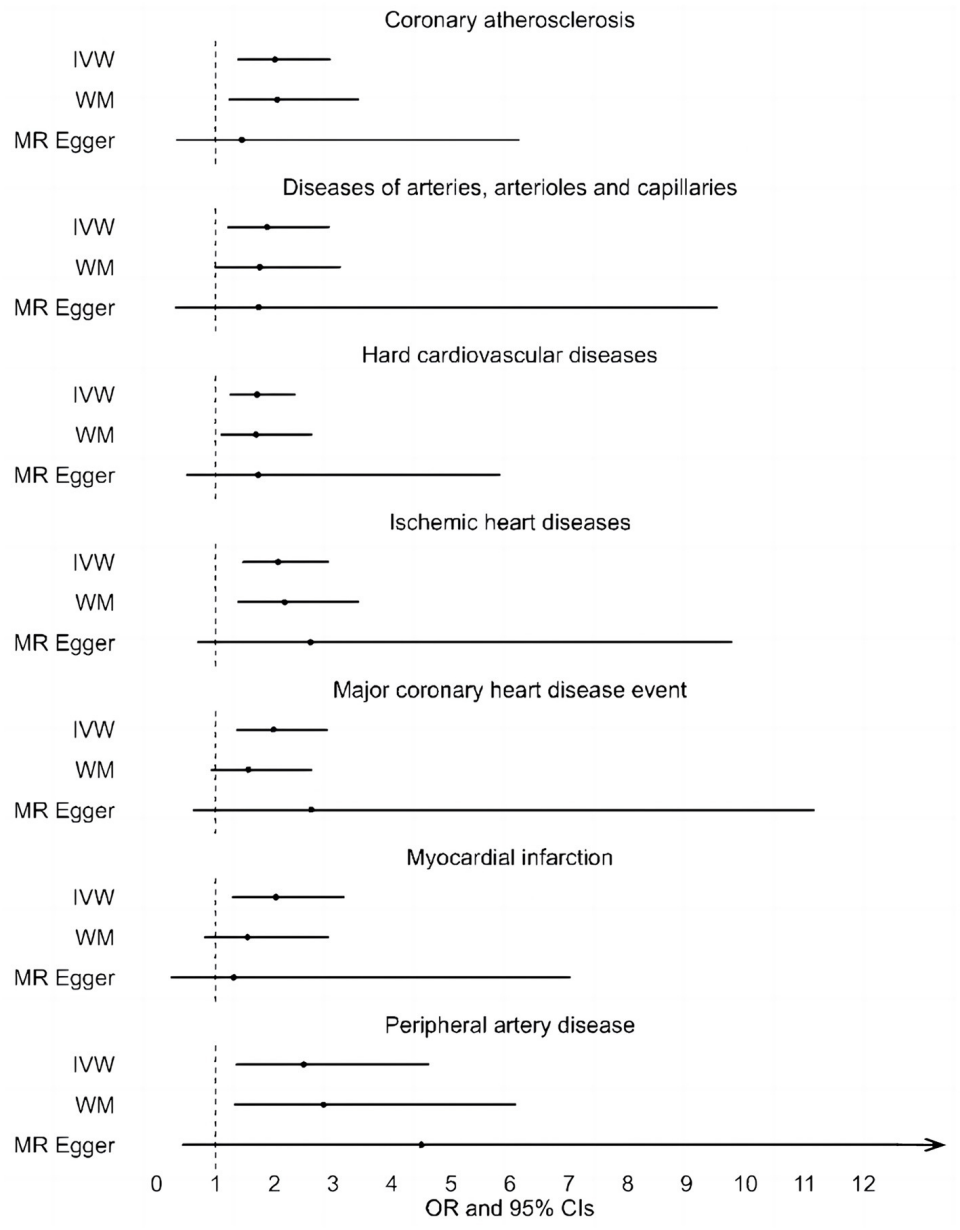
Associations between genetically predicted U_Na_/U_Cr_ and seven CVDs examined by three MR methods. MR, Mendelian randomization; U_Na_/U_Cr_, urinary sodium/creatinine ratio; IVW, inverse-variance weighted; WM, weighted median; OR, odds ratio; CI, confidence interval.

**Figure 3:**
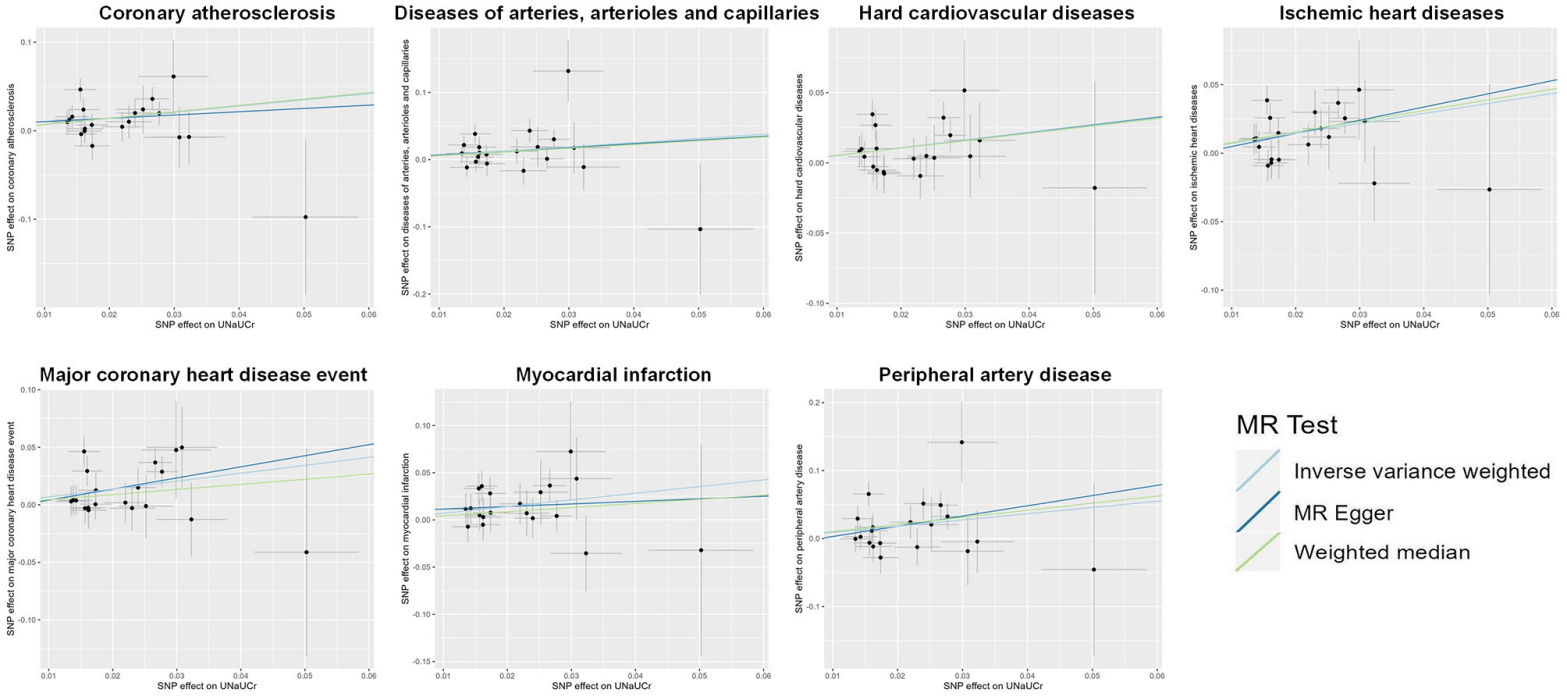
Scatter plot showing the causal effects of U_Na_/U_Cr_ on seven CVDs. SNP, single-nucleotide polymorphism; U_Na_/U_Cr_, urinary sodium/creatinine ratio.

**Figure 4:**
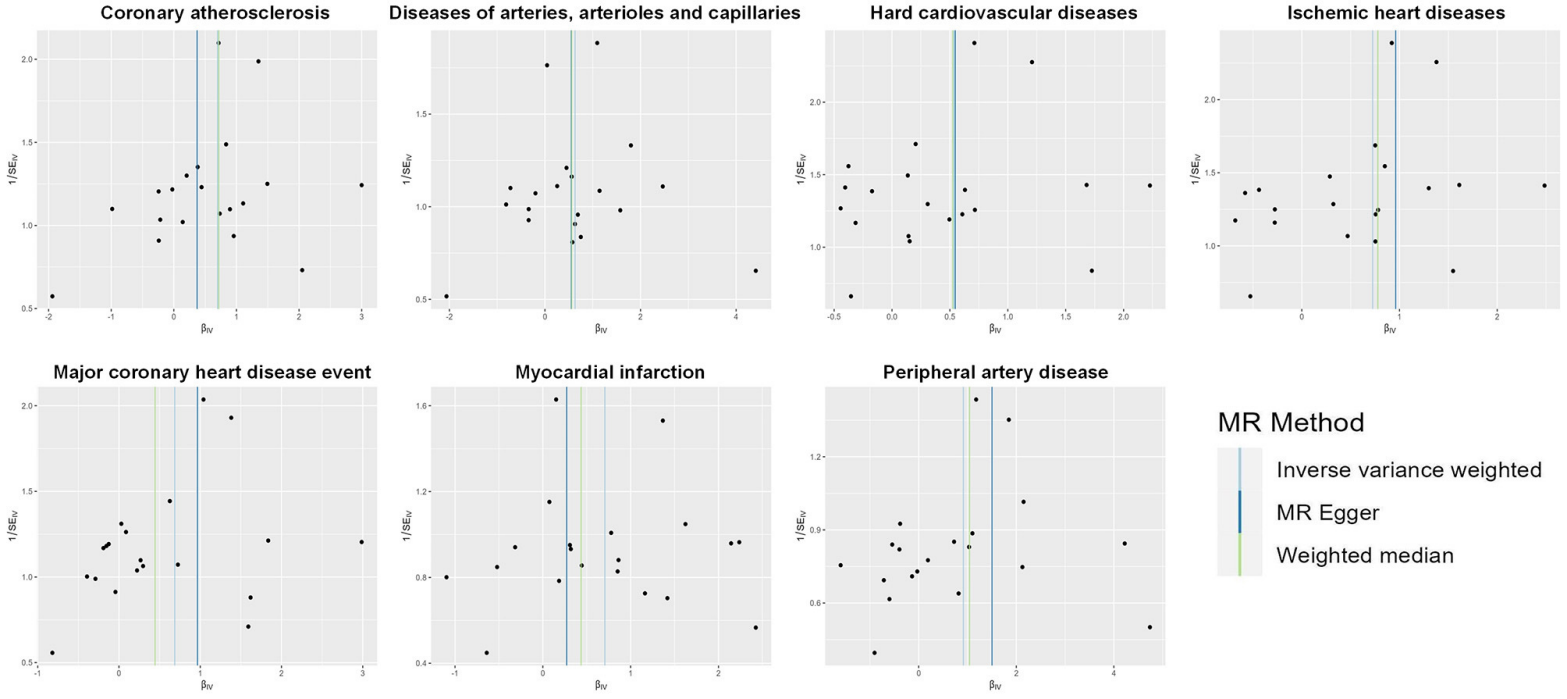
Funnel plot indicating the causal associations of U_Na_/U_Cr_ on seven CVDs. SNP, single-nucleotide polymorphism; U_Na_/U_Cr_, urinary sodium/creatinine ratio; IV, instrumental variable; SE, standard error.

**Figure 5:**
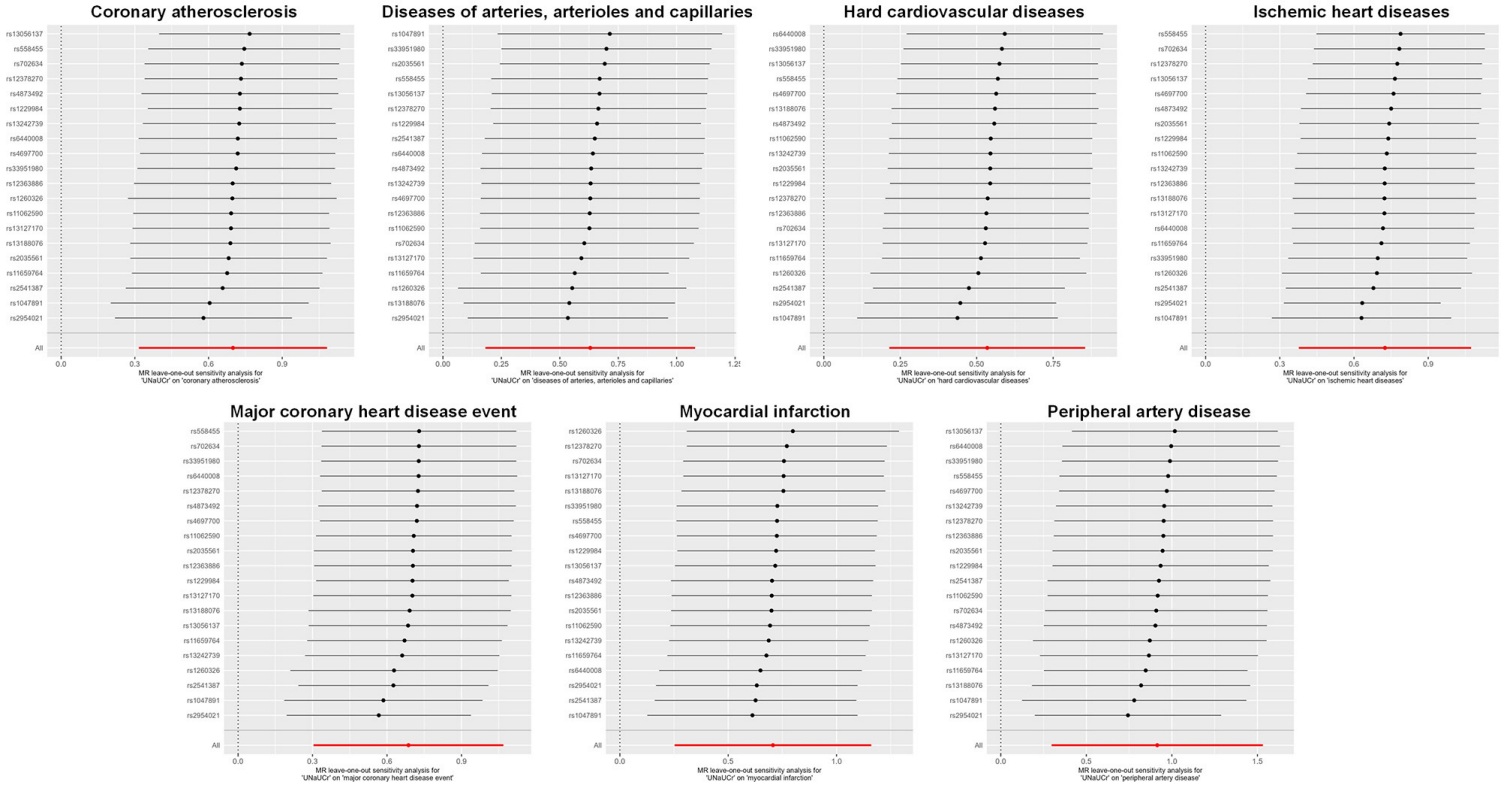
Leave-one-out sensitivity analysis examining the causal estimates of U_Na_/U_Cr_ on seven CVDs by the IVW method after excluding a specific SNP from the analysis. MR, Mendelian randomization; SNP, single-nucleotide polymorphism; U_Na_/U_Cr_, urinary sodium/creatinine ratio; IVW, inverse-variance weighted.

A total of 20 distinct SNPs were used as the genetic IVs for U_Na_/U_Cr_, and the F-statistics for the IVs ranged from 30.53 to 125.48, suggesting good instrument strength (Supplementary Table 2). FDR correction for multiple testing was used to guide the interpretation of the findings. The genetically predicted U_Na_/U_Cr_ was found to be strongly linked with 7 of 50 CVDs, according to the IVW approach in MR, including “Coronary atherosclerosis” (OR = 2.01; 95% CI: 1.37, 2.95), “Diseases of arteries, arterioles and capillaries” (OR = 1.88; 95% CI: 1.20, 2.94), “Hard cardiovascular diseases” (OR = 1.71; 95% CI: 1.24, 2.35), “Ischemic heart diseases” (OR = 2.06; 95% CI: 1.46, 2.93), “Major coronary heart disease event” (OR = 1.99; 95% CI: 1.36, 2.91), “Myocardial infarction” (OR = 2.03; 95% CI: 1.29, 3.19), and “Peripheral artery disease” (OR = 2.50; 95% CI: 1.35, 4.63) (Figures 1, 2; Supplementary Table 3). Using the MR-Egger and WM approaches, the relationships between U_Na_/U_Cr_ and seven CVDs had the same direction (Figure 2; Supplementary Table 3). The scatter plot visually showed causal associations between U_Na_/U_Cr_ and seven diseases of the circulation system (Figure 3). This analysis revealed no discernible heterogeneity (Figure 4; Supplementary Table 4). According to the intercept term of the MR-Egger technique (Supplementary Table 5), horizontal pleiotropy was not significant in the causality analysis, which is similar to the findings of MR-PRESSO, where no outlier IV was found. The leave-one-out analysis revealed that the exclusion of one SNP could not significantly change the observed outcomes (Figure 5).

## Discussion

This study employed a two-sample MR analysis to evaluate the causal association between sodium intake and a wide range of 50 CVDs, leveraging data from public databases and comprehensive GWAS. The results showed a positive causal relationship between U_Na_/U_Cr_ and seven types of CVDs. To account for the overlap among some of the seven CVDs, we have categorized and discussed them separately into the following two categories.

### The site of disease onset is primarily in the heart

Ischemic heart disease (IHD) is an umbrella term used to describe various heart problems resulting from reduced blood flow to the heart. IHD is the predominant manifestation of coronary heart diseases (CHDs). It arises due to arterial stenosis, or the narrowing of the blood vessels responsible for supplying oxygenated blood to the myocardium, also known as the heart muscle (22). A model study conducted in China demonstrated that reducing salt intake by 1 gram per day could potentially lower the risk of IHD by approximately 4% (23). The findings of our study are consistent with prior research. IHD is most commonly caused by atherosclerosis (24, 25). A Swedish cohort study found a correlation between a higher estimated 24-h sodium excretion and coronary atherosclerosis (26). This finding provides additional support for the conclusions drawn in our study. Major coronary heart disease (CHD) events consist of both fatal and non-fatal events, with the former defined as death resulting from CHD and the latter indicating myocardial infarction or heart attack (27). Several observational studies have found that high urinary sodium excretion is linked to an increased risk of myocardial infarction. For example, a study carried out in China revealed a significant correlation between excessive excretion of urinary sodium and increased susceptibility to cardiovascular diseases. This connection was noticed concerning instances of non-fatal heart attacks and fatalities attributed to coronary heart disease (28). Additionally, a meta-analysis indicated that elevated sodium levels, detected through multiple 24-h urine collections, were associated with a heightened risk of cardiovascular events. These occurrences encompass both lethal and non-lethal instances of heart attack, stroke, or the requirement for coronary revascularization (29).

Arterial stiffness is a well-known contributor to CVD. Studies have found that excessive sodium intake in the diet can induce changes in the extracellular matrix of the arterial wall, thereby facilitating the process of arteriosclerosis (30). Arterial stiffness is characterized by an imbalance between elastin and collagen fibers, which are key components of arterial elasticity. This process is regulated by matrix metalloproteinases (MMPs) (31). It has been discovered that a high sodium intake leads to the activation of extracellular matrix metalloproteinases, specifically MMP-2 and MMP-9, which subsequently stimulate the production of transforming growth factor-beta 1 (TGF-β1) (31, 32). TGF-β1 is a fibrotic growth factor that, when overexpressed, can cause thickening of the arterial intima, thinning and fracturing of elastin fibers, and a reduction in the ratio of elastin to collagen. These changes ultimately contribute to the development of arterial stiffness (33).

Furthermore, an MR study investigating the impact of urinary sodium on cardiovascular risk factors, ischemic stroke, and heart failure (HF) demonstrated that higher levels of urinary sodium were associated with an elevated risk of both HF and global ischemic stroke (34). Consistent with our own findings, a cohort study conducted in Taiwan revealed a significant association between increased urine sodium excretion and a higher risk of CVD, particularly stroke (35). However, a Finnish cohort study reported contrasting results, finding no significant correlation between urinary sodium levels and major adverse coronary events in men with HF (36). This discrepancy may be attributed to the population selection in the study, which excluded female patients with chronic HF and included only male patients.

### The onset site is not within the heart

This study has shown a positive causal association between U_Na_/U_Cr_ and peripheral arterial disease, as well as diseases affecting arteries, arterioles, and capillaries. These findings are in line with our study and support the conclusions of previous investigations. Two cross-sectional studies conducted in China demonstrated a robust positive association between urine sodium excretion and the presence of carotid atherosclerosis (37, 38). In a Korean cohort study, it was found that dietary sodium intake in adults aged 40 years and older may be positively associated with subsequent levels of carotid intima-media thickness (39). However, another cross-sectional study and systematic evaluation reported a limited correlation between small vessel disease and current salt intake, or U_Na_/U_Cr_ (40).

The underlying biological mechanism may be due to oxidative stress and endothelial dysfunction. The endothelium serves as a dynamic monolayer that critically regulates vascular tone and inflammation in the blood vessel wall (41, 42). Endothelial cells synthesize nitric oxide (NO), which acts as a protective molecule crucial for maintaining optimal vascular function (43, 44). Several enzymatic systems, such as NADPH oxidase and xanthine oxidase, are responsible for deactivating NO while simultaneously increasing levels of superoxide anions (${\text{O}}_{2}^{•-}$) (45), contributing to the development of endothelial dysfunction. Previous studies have demonstrated that salt loading impairs vascular endothelial function (46, 47). In a study on middle-aged hypertensive individuals, restricted sodium intake was found to reverse microvascular endothelial dysfunction through a reduction in oxidative stress (48). Additionally, administration of the antioxidant ascorbic acid improved microvascular function after another sodium-induced impairment, further supporting the role of oxidative stress in this process (49).

## Strengths and limitations

This study has several noteworthy advantages. First, a two-sample MR strategy was used in the study to reduce the influence of confounding factors and reverse causality on the outcomes. Second, this study is the largest investigation to date on the causal relationship between U_Na_/U_Cr_ and a broad type of CVD. Finally, we achieved relatively robust results by using GWAS summary statistics and conducting various sensitivity analyses to minimize the possibility of horizontal pleiotropy.

Our study also has limitations. First, our study only included individuals from Europe, which limits the generalizability of our findings to other ethnic groups because there may be variations in the association between U_Na_/U_Cr_ and CVD across different ethnicities. Further research should encompass large-scale GWAS across diverse geographical regions. Second, selection bias may have affected our results, as individuals who died due to outcome competing risk might have been missed in GWAS. Third, stratification is necessary when populations demonstrate different disease rates, trait distributions, and allele frequencies. However, we encountered limitations in conducting population stratification based on additional factors such as age and gender due to using GWAS summary data instead of individual-level data in our study. Finally, previous observational studies have shown a U-shaped or J-shaped relationship between sodium intake and diverse cardiovascular diseases (50, 51), and this study neglects assessing possible non-linear connections between sodium consumption and outcomes. Future research utilizing extensive biobanks may provide further insight into the potential existence of such a relationship.

## Conclusion

Our research has identified a significant and positive causal relationship between U_Na_/U_Cr_ and seven CVDs. This provides evidence that elevated levels of sodium intake may play a contributing role in the onset or advancement of these CVDs. Therefore, it underscores the importance of closely monitoring and effectively managing sodium intake to mitigate the risk of developing CVD.

## Data availability statement

The original contributions presented in the study are included in the article/Supplementary material, further inquiries can be directed to the corresponding authors.

## Ethics statement

Ethical review and approval was not required for the study on human participants in accordance with the local legislation and institutional requirements. Written informed consent from the patients/participants or patients/participants' legal guardian/next of kin was not required to participate in this study in accordance with the national legislation and the institutional requirements.

## Author contributions

SL designed the manuscript. QF, RC, YD, SX, CH, BH, TJ, and BZ performed the statistical analyses and drafted the manuscript. MB critically reviewed the manuscript. All authors read and approved the final manuscript.
