# Supplementary material for: Sodium intake and the risk of various types of cardiovascular diseases: a Mendelian randomization study
Source: Front Nutr. 2023 Dec 22;10:1250509. doi: 10.3389/fnut.2023.1250509 (PMC10771828; doi:10.3389/fnut.2023.1250509)
Supplement: Supplementary file 1 [file Data_Sheet_1.docx]

**Supplementary Table 1**. Information of GWAS summary datasets used in MR analyses.

| Traits | Consortium | Phenotype name | Sample size |
| --- | --- | --- | --- |
| U_Na_/U_Cr_ | UKB | urinary sodium/creatinine ratio | 327616 |
| All-cause Heart Failure | FinnGen | I9_HEARTFAIL_ALLCAUSE | 218208 |
| Angina pectoris | FinnGen | I9_ANGINA | 206008 |
| Aortic aneurysm | FinnGen | I9_AORTANEUR | 209366 |
| Arterial embolism and thrombosis | FinnGen | I9_ARTEMBTHR | 207330 |
| Atrial fibrillation and flutter | FinnGen | I9_AF | 138994 |
| AV-block | FinnGen | I9_AVBLOCK | 159099 |
| Cardiac arrest | FinnGen | I9_CARDARR | 118055 |
| Cardiomyopathy | FinnGen | I9_CARDMYO | 159811 |
| Cardiovascular diseases | FinnGen | I9_CVD | 218792 |
| Cerebral aneurysm, nonruptured | FinnGen | I9_ANEURYSM | 204060 |
| Cerebral atherosclerosis | FinnGen | I9_CEREBATHER | 218792 |
| Cerebrovascular diseases | FinnGen | I9_CEREBVASC | 218792 |
| Conduction disorders | FinnGen | I9_CONDUCTIO | 161127 |
| Coronary atherosclerosis | FinnGen | I9_CORATHER | 211203 |
| Death due to cardiac causes | FinnGen | I9_K_CARDIAC | 218792 |
| Deep vein thrombosis of lower extremities | FinnGen | I9_PHLETHROMBDVTLOW | 194604 |
| Diseases of arteries, arterioles and capillaries | FinnGen | I9_DOAAC | 218792 |
| Dissection of aorta | FinnGen | I9_AORTDIS | 207011 |
| Dissection of cerebral arteries, nonruptured | FinnGen | I9_DISCER | 203163 |
| Endocarditis | FinnGen | I9_ENDOCARD | 157164 |
| Hard cardiovascular diseases | FinnGen | I9_CVD_HARD | 218792 |
| Heart failure,strict | FinnGen | I9_HEARTFAIL | 208178 |
| Hypertension | FinnGen | I9_HYPTENS | 218754 |
| Hypertrophic cardiomyopathy | FinnGen | I9_HYPERTROCARDMYOP | 218792 |
| Hypotension | FinnGen | I9_HYPOTE | 218320 |
| Intracerebral haemmorrhage | FinnGen | I9_ICH | 202833 |
| Ischaemic Stroke | FinnGen | I9_STR_EXH | 212774 |
| Ischemic heart diseases | FinnGen | I9_ISCHHEART | 218792 |
| Major coronary heart disease event | FinnGen | I9_CHD | 218792 |
| Myocardial infarction | FinnGen | I9_MI_STRICT | 199462 |
| Myocarditis | FinnGen | I9_MYOCARD | 117755 |
| Nonpyogenic thrombosis of intracranial venous system | FinnGen | I9_THROMBICV | 203176 |
| Non-rheumatic valve diseases | FinnGen | I9_NONRHEVALV | 166946 |
| Nontraumatic intracranial haemmorrhage | FinnGen | I9_INTRACRA | 205862 |
| Oesophageal varices | FinnGen | I9_VARICVEOES | 190513 |
| Paroxysmal tachycardia | FinnGen | I9_PAROXTAC | 121713 |
| Pericarditis | FinnGen | I9_PERICARD | 157195 |
| Peripheral artery disease | FinnGen | I9_PAD | 213639 |
| Phlebitis and thrombophlebitis | FinnGen | I9_PHLETHROM | 193305 |
| Portal vein thrombosis | FinnGen | I9_PVT | 190183 |
| Progressive vascular leukoencephalopathy | FinnGen | I9_PROGVASC | 203338 |
| Pulmonary embolism | FinnGen | I9_PULMEMB | 218413 |
| Pulmonary heart disease | FinnGen | I9_PULMHEART | 218792 |
| Rheumatic valve diseases | FinnGen | I9_RHEUVALV | 218623 |
| Stroke | FinnGen | I9_STR | 212884 |
| Subarachnoid haemmorrhage | FinnGen | I9_SAH | 202568 |
| Transient ischemic attack | FinnGen | I9_TIA | 211058 |
| Unstable angina pectoris | FinnGen | I9_UAP | 204688 |
| Varicose veins | FinnGen | I9_VARICVE | 207055 |
| Venous thromboembolism | FinnGen | I9_VTE | 218792 |
| Abbreviation: GWAS: genome-wide association study; MR: Mendelian randomization; U_Na_/U_Cr_: urinary sodium/creatinine ratio; UKB: UK Biobank.  The definition of phenotypes can be found at https://risteys.finregistry.fi. | | | |

**Supplementary Table 2**. The F-statistics of IVs.

| SNP | EA | OA | 1. Statistic |
| --- | --- | --- | --- |
| rs1047891 | A | C | 104.83 |
| rs11062590 | G | C | 35.60 |
| rs11659764 | A | T | 30.66 |
| rs1229984 | C | T | 37.93 |
| rs12363886 | A | C | 30.53 |
| rs12378270 | A | G | 33.68 |
| rs1260326 | C | T | 125.48 |
| rs13056137 | A | C | 40.01 |
| rs13127170 | G | A | 31.91 |
| rs13188076 | T | G | 69.83 |
| rs13242739 | A | T | 31.17 |
| rs2035561 | C | T | 30.53 |
| rs2541387 | C | A | 42.56 |
| rs2954021 | G | A | 41.23 |
| rs33951980 | T | C | 41.11 |
| rs4697700 | C | G | 32.54 |
| rs4873492 | T | C | 46.20 |
| rs558455 | G | A | 40.83 |
| rs6440008 | C | T | 47.83 |
| rs702634 | A | G | 38.13 |
| Abbreviation: IVs: instrumental variables; SNP: single nucleotide polymorphism; EA: effect allele; OA: other allele. | | | |

**Supplementary Table 3**. MR analysis results.

| Outcome | Method | Number of SNPs | OR | 95% CI | *P* |
| --- | --- | --- | --- | --- | --- |
| Coronary atherosclerosis | IVW | 20 | 2.01 | 1.37-2.95 | 3.45E-04 |
| Coronary atherosclerosis | MR-Egger | 20 | 1.45 | 0.34-6.16 | 6.21E-01 |
| Coronary atherosclerosis | WM | 20 | 2.05 | 1.21-3.47 | 7.42E-03 |
| Diseases of arteries, arterioles and capillaries | IVW | 20 | 1.88 | 1.20-2.94 | 5.86E-03 |
| Diseases of arteries, arterioles and capillaries | MR-Egger | 20 | 1.74 | 0.32-9.53 | 5.34E-01 |
| Diseases of arteries, arterioles and capillaries | WM | 20 | 1.75 | 0.99-3.10 | 5.30E-02 |
| Hard cardiovascular diseases | IVW | 20 | 1.71 | 1.24-2.35 | 1.06E-03 |
| Hard cardiovascular diseases | MR-Egger | 20 | 1.73 | 0.51-5.83 | 3.91E-01 |
| Hard cardiovascular diseases | WM | 20 | 1.69 | 1.08-2.65 | 2.19E-02 |
| Ischemic heart diseases | IVW | 20 | 2.06 | 1.46-2.93 | 4.52E-05 |
| Ischemic heart diseases | MR-Egger | 20 | 2.61 | 0.70-9.78 | 1.70E-01 |
| Ischemic heart diseases | WM | 20 | 2.18 | 1.36-3.49 | 1.23E-03 |
| Major coronary heart disease event | IVW | 20 | 1.99 | 1.36-2.91 | 4.31E-04 |
| Major coronary heart disease event | MR-Egger | 20 | 2.63 | 0.62-11.17 | 2.07E-01 |
| Major coronary heart disease event | WM | 20 | 1.56 | 0.92-2.64 | 9.86E-02 |
| Myocardial infarction | IVW | 20 | 2.03 | 1.29-3.19 | 2.27E-03 |
| Myocardial infarction | MR-Egger | 20 | 1.31 | 0.25-7.02 | 7.54E-01 |
| Myocardial infarction | WM | 20 | 1.55 | 0.82-2.92 | 1.79E-01 |
| Peripheral artery disease | IVW | 20 | 2.50 | 1.35-4.63 | 3.71E-03 |
| Peripheral artery disease | MR-Egger | 20 | 4.50 | 0.44-46.17 | 2.22E-01 |
| Peripheral artery disease | WM | 20 | 2.83 | 1.31-6.15 | 8.39E-03 |
| Abbreviation: MR: Mendelian randomization; OR: odds ratio; CI: confidence interval; SNP: single nucleotide polymorphism; IVW: inverse-variance weighted; WM: weighted median. | | | | | |

**Supplementary Table** **4**. Heterogeneity test results.

| Outcome | Q | Q_df | Q_*P* |
| --- | --- | --- | --- |
| Coronary atherosclerosis | 22.61 | 19 | 0.255 |
| Diseases of arteries, arterioles and capillaries | 24.72 | 19 | 0.170 |
| Hard cardiovascular diseases | 20.81 | 19 | 0.348 |
| Ischemic heart diseases | 24.13 | 19 | 0.191 |
| Major coronary heart disease event | 21.20 | 19 | 0.326 |
| Myocardial infarction | 13.66 | 19 | 0.803 |
| Peripheral artery disease | 27.37 | 19 | 0.096 |

**Supplementary Table 5**. MR-Egger pleiotropy test results.

| Outcome | Egger_intercept | SE | *P* |
| --- | --- | --- | --- |
| Coronary atherosclerosis | 0.0066 | 0.0143 | 0.650 |
| Diseases of arteries, arterioles and capillaries | 0.0016 | 0.0169 | 0.926 |
| Hard cardiovascular diseases | -0.0002 | 0.0121 | 0.985 |
| Ischemic heart diseases | -0.0048 | 0.0131 | 0.720 |
| Major coronary heart disease event | -0.0056 | 0.0143 | 0.699 |
| Myocardial infarction | 0.0088 | 0.0166 | 0.604 |
| Peripheral artery disease | -0.0119 | 0.0231 | 0.613 |
| Abbreviation: MR: Mendelian randomization; SE: standard error. | | | |
